# The soil mite genus *Conchogneta* (Acari, Oribatida, Autognetidae), with new findings from Mongolia

**DOI:** 10.3897/zookeys.178.2758

**Published:** 2012-03-29

**Authors:** Badamdorj Bayartogtokh

**Affiliations:** 1 Department of Zoology, School of Biology and Biotechnology, National University of Mongolia, Ulaanbaatar 210646, Mongolia

**Keywords:** Oribatida, *Conchogneta*, biogeography, habitat ecology, new species, Mongolia

## Abstract

This work deals with taxonomy, geographical distribution as well as known ecology of oribatid mites of the genus *Conchogneta* Grandjean, 1963 in the world. The majority of species belonging to this genus is known to be widely distributed in Europe, but only three of them are found in other areas of the northern hemisphere. Most species of *Conchogneta* are inhabitants of litter of various types of forestas, terricolous and epiphytic bryophytes, epiphytic lichens, and soil of steppe, river valleys, moor, oligotrophic bogs, floodland assemblages etc. A new species, *Conchogneta glabrisensillata* **sp. n.** is described, and another species, *Conchogneta traegardhi* (Forsslund, 1947) is redescribed from the northern and western parts of Mongolia, respectively. *Conchogneta* is recorded for the first time for the fauna of Mongolia. The species status of *Conchogneta dalecarlica* (Forsslund, 1947) is discussed. Species descriptions are accompanied with detailed illustrations. Furthermore, a key is provided for the identification of adults of the known species of *Conchogneta* in the world.

## Introduction

The oribatid mite genus *Conchogneta* is one of seven genera in the family Autognetidae, which was erected by Grandjean (1963) with the type species, *Autogneta dalecarlica* Forsslund, 1947. Currently, the genus comprises seven nominal species and one subspecies, the majority of which have restricted distributions in the Palaearctic region, especially in Europe. Two eastern European species, *Conchogneta vasiliorum* Mahunka, 2006 and *Conchogneta weigmanni* Mahunka, 2007 are known until today only from Romania; another species, *Conchogneta inundata* (Winkler, 1957) is reported from the Czech Republic; *Conchogneta willmanni herzegowinensis* (Willmann, 1941) is known from Bosnia and Herzegovina. Three other species, *Conchogneta traegardhi* (Forsslund, 1947), *Conchogneta willmanni* (Dyrdowska, 1929) and *Conchogneta dalecarlica* (Forsslund, 1947) have rather wide distributions in the Palaearctic or even Holarctic region, and all these three species were recorded in Europe and Asia; *Conchogneta traegardhi* (Forsslund, 1947) was also found in North America. *Conchogneta iranica* Akrami, 2008 is the single species which is only known from Asia (Dyrdowska 1929, Willmann 1941, Forsslund 1947, Winkler 1957, Grandjean 1963, Woas 1986; Marshall et al. 1987, Mahunka 2006, 2007, Akrami 2008, Toluk and Ayyildiz 2009).

The genus *Conchogneta* is unique among other genera of Autognetidae in the combination of following characters: rostrum with deep medial incision; prodorsal costulae long, mostly medially positioned close to each other, but rarely distantly placed laterally from each other; sensilli narrow, setiform or with dilated head; anterior part of notogaster without crista; tibia I with large dorso-distal tubercle overhanging tarsus I.

The immatures of *Conchogneta* are apheredermous, which means nymphs (and adults) do not retain scalps, unideficient, and have setae *d* on tibiae and genua of legs when respective solenidia exist. However, the morphology of immatures of most *Conchogneta* species is poorly known, and only two of them, such as *Conchogneta dalecarlica* and *Conchogneta traegardhi* are studied in terms of juvenile morphology and patterns of their postembryonic development (Grandjean 1963, Ermilov and Łochynska 2009, Ermilov 2011).

The aim of this work is to describe an unknown species, *Conchogneta glabrisensillata* sp. n., and redescribe another recently collected species, *Conchogneta traegardhi* (Forsslund, 1947) from northern and western Mongolia, respectively. The latter species is recorded for the first time in Mongolia. A review of the composition of genus *Conchogneta* with remarks on the biogeography and habitat ecology of its members, and a wold-wide identification key to *Conchogneta* are additionally provided. The taxonomic status of *Conchogneta dalecarlica* is discussed, which was argued previously in different literature. The study of oribatid mite diversity in Mongolia is the subject of ongoing research as part of the biodiversity assessments in various habitats of the country with emphasizes of the effects of climate change and influence of pastoral livestock grazing.

## Material and methods

All materials used in this study were collected by the author with assistance of some of his graduate students and specimens were mounted in temporary slides to view the anterior, lateral and posterior aspects, and then preserved in alcohol. All examined materials and data on their localities are given in the respective ‘material examined’ section. Species studied here are represented as adults.

Specimens were cleared in lactic acid, and a differential interference contrast microscope was used for investigation in transmitted light. Line drawings were made using a camera lucida attached to the compound microscope. Micrographs were taken using a digital camera (Olympus Altra 20) attached to the microscope with single shot.

The morphological terminology used below is mostly that developed over many years by Grandjean (1960a, b, 1963), and also that by Lions (1975), Norton and Behan-Pelletier (2009). All measurements are given as a range, with the mean in parentheses. Body length was measured in lateral view, from the tip of the rostrum to the posterior edge of the ventral plate, to avoid discrepancies caused by different degrees of notogastral distension. Notogastral length was also measured in lateral aspect (when the dorsosejugal groove is discernable), from the anterior to the posterior edge; notogastral width refers to the maximum width in dorsal aspect. Setal formulas of the legs are given as numbers per segment for appendages (from trochanter to tarsus) and as number per podosomal segment (I-IV) for epimeres. Most species of Autognetidae show the same structure and setation of legs, palps and chelicerae. Therefore, in this work I made detailed descriptions and illustrations of the chelicera and the palp only for one of the studied species.

## Results

### Conchogneta glabrisensillata sp. n.

urn:lsid:zoobank.org:act:338C1B69-2EFB-4D90-B98F-2289E32773ED

http://species-id.net/wiki/Conchogneta_glabrisensillata

Figs 1 [Fig F2] –3

#### Diagnosis.

Medium in size (378–427 μm in length); rostrum with deep incision reaching level of rostral setal insertion; prodorsal costula long, slightly sigmoid, diverging proximally, but converging medially and again very slightly diverging anteriorly; sensillus smooth, with relatively long stalk and slender, lanceolate head; rostral seta barbed, lamellar and interlamellar setae smooth; prodorsal tubercles *Ea* small, *Ep* large; interbothridial region with one pair of tubercles; exobothridial region with small granular tubercles; notogastral setae long, thin.

#### Measurements.

Holotype: body length 384 μm, length of notogaster 256 μm, width of notogaster 201 μm; paratypes (*n* = 3) body length 378–427 (405) μm; length of notogaster 250–281 (266) μm; width of notogaster 192–213 (204) μm.

*Integument.* Body color yellowish brown to light brown. Surface of body and leg segments with very thin, nearly smooth cerotegument. Integument microtuberculate on tubercles, prodorsum, lateral part of prodorsum, notogaster and around leg acetabula.

**Figure 1.**
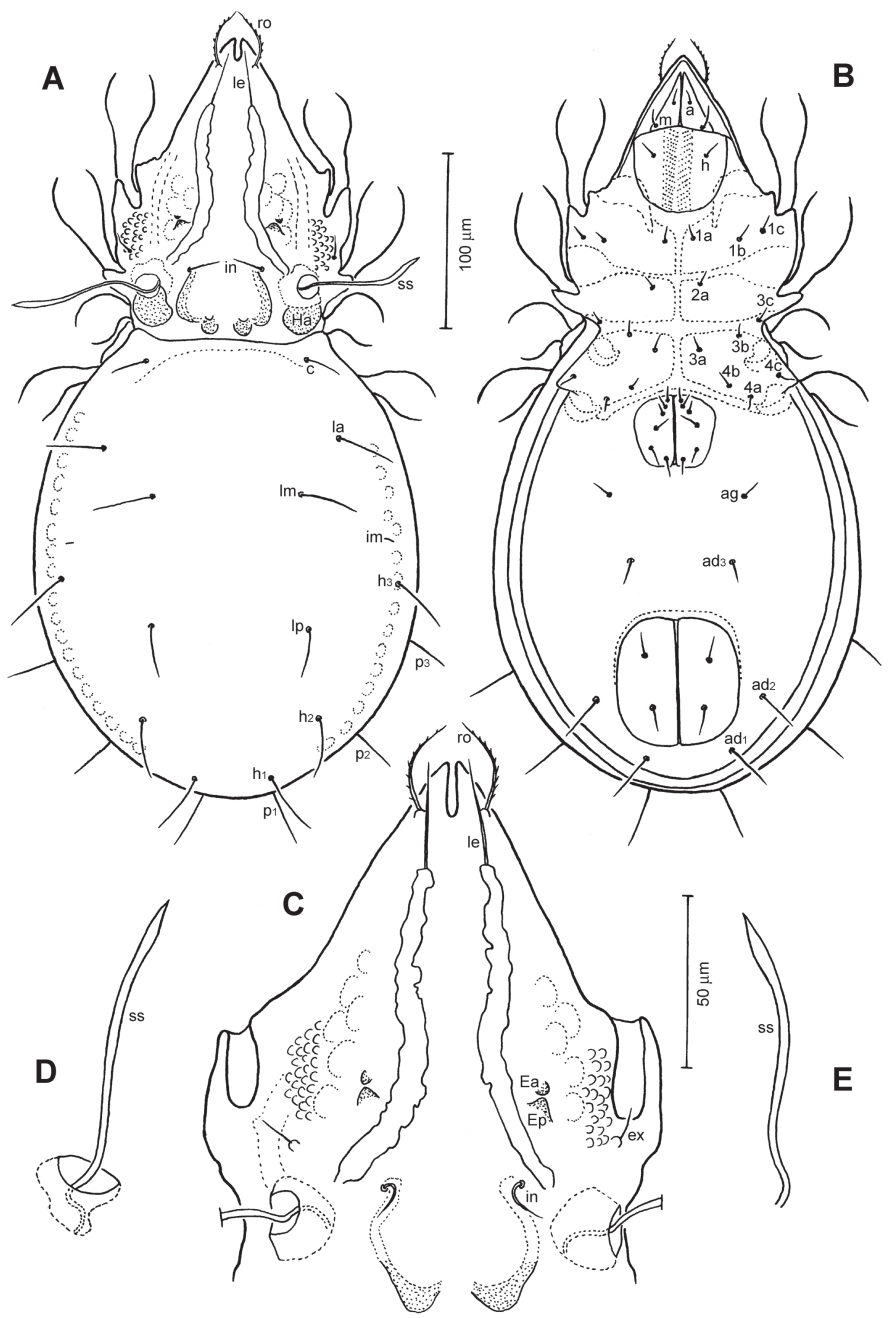
*Conchogneta glabrisensillata* sp. n. **A** Dorsal view of idiosoma **B** Ventral view of idiosoma **C** Prodorsum **D** Sensillus and bothridium, lateral view **E** Slight variation of sensillus, lateral view.

**Figure 2. F2:**
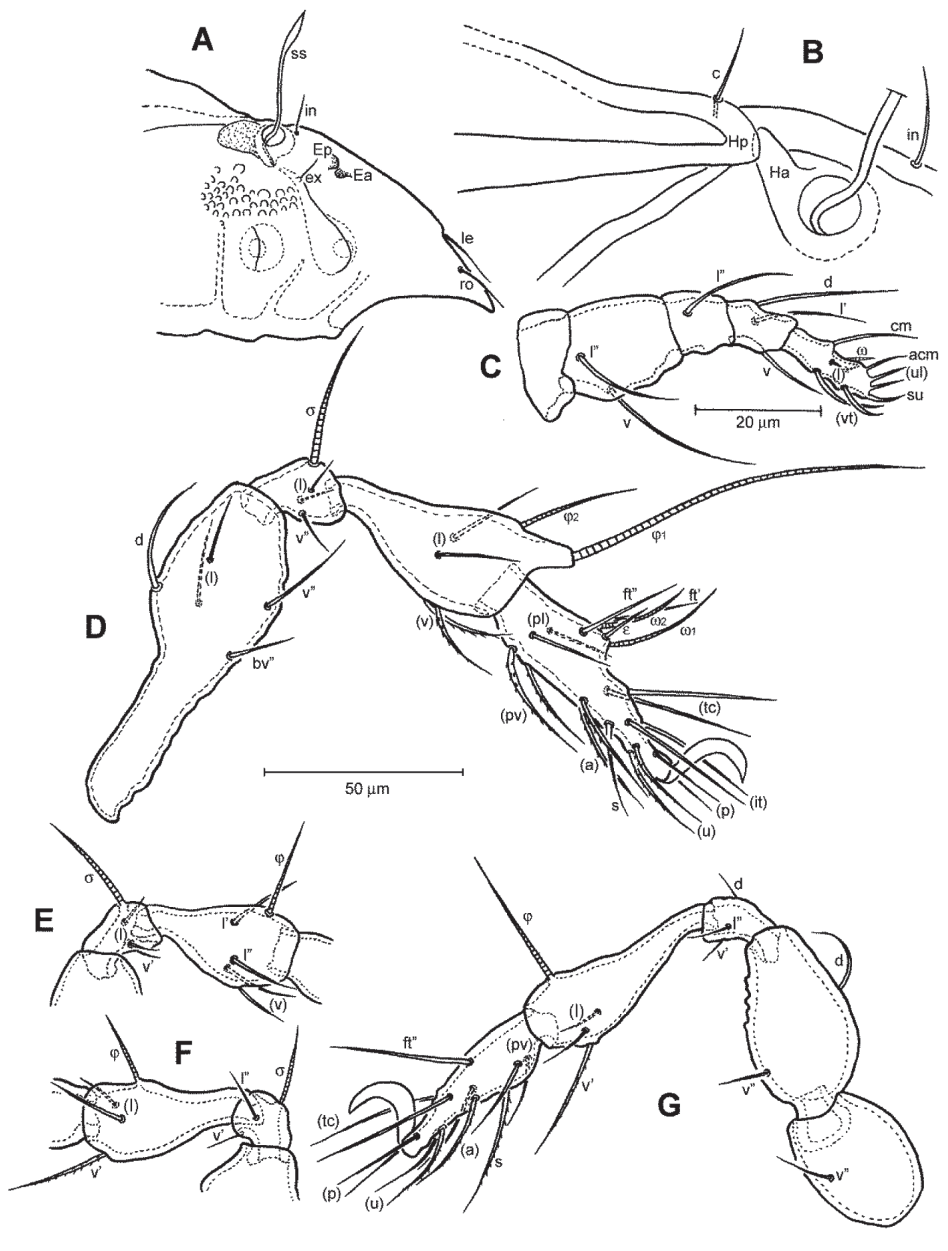
*Conchogneta glabrisensillata* sp. n. **A** Lateral view of prodorsum and anterior part of notogaster **B** Humeral region, showing tubercles *Ha* and *Hp* **C** Palp, right, antiaxial view **D** Leg I, right, antiaxial view **E** Genu and tibia of leg II, right, antiaxial view **F** Genu and tibia of leg III, right, antiaxial view **G** Leg IV, right, antiaxial view.

**Figure 3.**
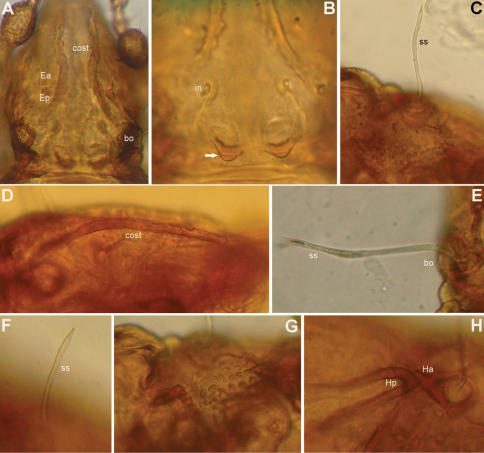
*Conchogneta glabrisensillata* sp. n. **A** Prodorsum, showing enantiophysis *E*, costula and bothridium **B** Central part of prodorsum, showing alveolus of interlamellar seta and interbothridial tubercle (indicated by arrow) **C** Part of laterial view of prodorsum, showing sensillus and granular tubercles on humeral region **D** Lateral view of prodorsal costula **E** Sensillus, lateral view **F** Slight variation of sensillus, lateral view **G** Granular tubercles on lateral part of prodorsum **H** Humeral region, showing tubercles *Ha* and *Hp*.

*Prodorsum* (Figs 1A, C, D, 2A, B, 3A, B). Rostrum with deep U-shaped incision reaching level of rostral setal insertion in dorsal view, but distinctly projecting anteroventrally in lateral view (Figs 1A, D, 2A). Rostral seta (*ro*) 30–36 μm long, barbed, curved medially, inserted dorsally on distinct tubercle. Prodorsal costula long, slightly sigmoid, diverging proximally, but converging medially and again very slightly diverging anteriorly (Figs 1C, 3A, D). Lamellar seta (*le*) thin, smooth, 31–38 μm long, straight, inserted at distal end of costula. Interlamellar seta (*in*) 15–21 μm long, attenuate, smooth; distance between alveoli of *in-in* greater than that of *ro-ro* as viewed in dorsal aspect (Fig. 1A, D). Exobothridial seta (*ex*) inserted on distinct tubercle, 11–13 μm in length, smooth, directed anterolaterally.Sensillus (*ss*) with relatively long stalk and slender, smooth, lanceolate head; exposed portion of sensillus 70–83 μm in length (Figs 1D, E, 3C, E, F). Bothridium (*bo*) large, its opening directed posterolaterally, with large protuberance (tubercle *Ha*) posteriorly (Figs 1A, 2A, B, 3H). Prodorsal enantiophysis *E* well developed, *Ea* small, but well observable; *Ep* large, subtriangular in shape (Figs 1C, 2A, 3A). Interbothridial region with one pair of tubercle, nearly semicircular as viewed in dorsal aspect (Figs 1A, 3B).

*Notogaster* (Figs 1A, 2B). Oval, slightly narrowed anteriorly, about 1.3 times as long as wide. Anterior margin nearly straight, with large humeral protuberance (tubercle *Hp*; Figs 2B, 3H); posterior margin evenly rounded as viewed in dorsal aspect (Fig. 1A). Notogastral setae medium long (29–38 μm in length), thin, smooth, not reaching level of insertions of next setal row. Lyrifissure *im* well developed; other lyrifissures and opisthonotal gland opening not evident.

*Gnathosoma* (Figs 1B, 2C). Subcapitular mentum slightly wider than long, with minute microtubercles. Hypostomal setae *h*, *a* and *m* medium long, thin, smooth (Figs 1B). Chelicera typical for genus as shown in description of next species, slender with few slightly sclerotized blunt teeth; seta *cha* about 1.4 times as long as *chb*, both setae smooth; Trägårdh’s organ small, but distinctly developed. Palp typical for genus as shown in figure 2C, palpal setation: 0-2-1-3-10, including solenidion ω of tarsus.

*Epimeral region* (Fig. 1B). Pedotecta I and II well developed, covered with minute granules. Epimeral region nearly smooth; setal formula 3-1-3-3, all setae medium long, smooth. Discidium well developed, projected laterally of leg acetabulum IV.

*Anogenital region* (Fig. 1B). Genital aperture slightly widened anteriorly, anal aperture with same width throughout. Genital, aggenital, anal and adanal setae *ad_3_* medium long; two other adanal setae, *ad_1_* and *ad_2_* relatively long, but all ano-genital setae thin, smooth. Adanal lyrifissure not evident.

*Legs* (Fig. 2D-G). Dorsal surface of claws smooth, tibia I with large dorso-distal tubercle overhanging tarsus I. Formula of leg setation (including famulus) I (1-5-3-4-18), II (1-5-3-4-15), III (2-3-2-3-15); IV (1-2-3-3-12); formula of solenidia: I (1-2-2); II (1-1-2); III (1-1-0); IV (0-1-0). Homology of leg setae and solenidia showed in Table 1.

**Table 1.** Leg setation of *Conchogneta glabrisensillata* sp. n.

**Legs**	**Trochanter**	**Femur**	**Genu**	**Tibia**	**Tarsus**
I	v’	d, (l), bv”, v”	(l), v”, σ	(l), (v), φ_1_, φ_2_	(ft), (tc), (it), (p), (u), (a), s, (pv), (pl), ε, ω_1_, ω_2_
II	v’	d, (l), bv”, v”	(l), v’, σ	(l), (v), φ	(ft), (tc), (it), (p), (u), (a), s, (pv), ω_1_, ω_2_
III	v’, l’	d, v’, l’	l”, v’, σ	(l), v’, φ	(ft), (tc), (it), (p), (u), (a), s, (pv)
IV	v”	d, v”	d, l”, v’	(l), v’, φ	ft”, (tc), (p), (u), (a), s, (pv)

#### Material examined.

Holotype (female): Sevsuul valley, Eastern shore of the Lake Hövsgöl, District Khankh, Province Hövsgöl, litter of cool temperate larch forest (*Larix sibiricus* Ledebour, 1833), 51°16'N, 100°74'E, elevation 1680 m, 08 July 2007, Col. B. Bayartogtokh; three paratypes (females) same data as holotype. The holotype and one paratype are deposited in the collection of the Department of Zoology, National University of Mongolia, Ulaanbaatar, Mongolia, and two paratypes are in the collection of the Senckenberg Museum of Natural History, Goerlitz, Germany. All type specimens are preserved in alcohol.

#### Remarks.

Among the eight known species of *Conchogneta*, only two of them, namely *Conchogneta traegardhi* (Forsslund, 1947) and *Conchogneta vasiliorum* Mahunka, 2006 resemble the present new species in the closely situated structure of prodorsal costulae. However, both mentioned species are different from the new speciesin the barbed head of sensilli as opposed to smooth sensilli in *Conchogneta glabrisensillata* sp. n. Moreover they differ in conspicuously barbed notogastral setae in contrast to smooth setae in the new species, nearly straight and thinner prodorsal costulae rather than sigmoid, but thicker costulae in the new species, and different structure of prodorsal tubercles *Ea* and *Ep*.

The other species, such as *Conchogneta dalecarlica* (Forsslund, 1947), *Conchogneta inundata* (Winkler, 1957), *Conchogneta iranica* Akrami, 2008, *Conchogneta willmanni* (Dyrdowska, 1929), *Conchogneta willmanni herzegowiensis* (Willmann, 1941) and *Conchogneta weigmanni* Mahunka, 2007 are easily distinguishable from the new species by the widely spaced prodorsal costulae, and different structure of prodorsal enantiophyses *E*.

#### Etymology.

The specific epithet “*glabrisensillata*” refers to the smooth sensillus or bothridial seta in the new species.

### Conchogneta traegardhi

(Forsslund, 1947)

http://species-id.net/wiki/Conchogneta_traegardhi

Figs 4 [Fig F5] –6

Autogneta trägårdhi Forsslund 1947, p. 114, fig. 3a, b.
Autogneta traegardhi : Golosova 1975, p. 224, fig. 530.
Conchongeta traegardhi : Subías 2004, p. 109; Mahunka 2006, p. 68, figs. 12–14; Weigmann 2006, p. 318, fig. 167c-e.
Autogneta (Autogneta) traegardhi : Subías 2010, p. 195.

#### Diagnosis.

Medium in size (353–387 μm in length); rostrum with deep incision reaching level of rostral setal insertion; prodorsal costula long, nearly straight, diverging proximally, but parallel anteriorly; sensillus with relatively long stalk and lanceolate head with few barbs at distal part; rostral seta barbed, lamellar and interlamellar setae smooth; prodorsal tubercles *Ea* and *Ep* small, same in size; interbothridial region with one pair of tubercles; exobothridial region with small granular tubercles; notogastral setae long, thin.

#### Measurements.

Body length 353–387 (368) μm; length of notogaster 225–251 (236) μm; width of notogaster 186–205 (198) μm.

*Integument*.Body color yellowish brown to light brown. Surface of body and leg segments with very thin, nearly smooth cerotegument. Integument microtuberculate on tubercles, prodorsum, lateral part of prodorsum, notogaster and around leg acetabula.

**Figure 4.**
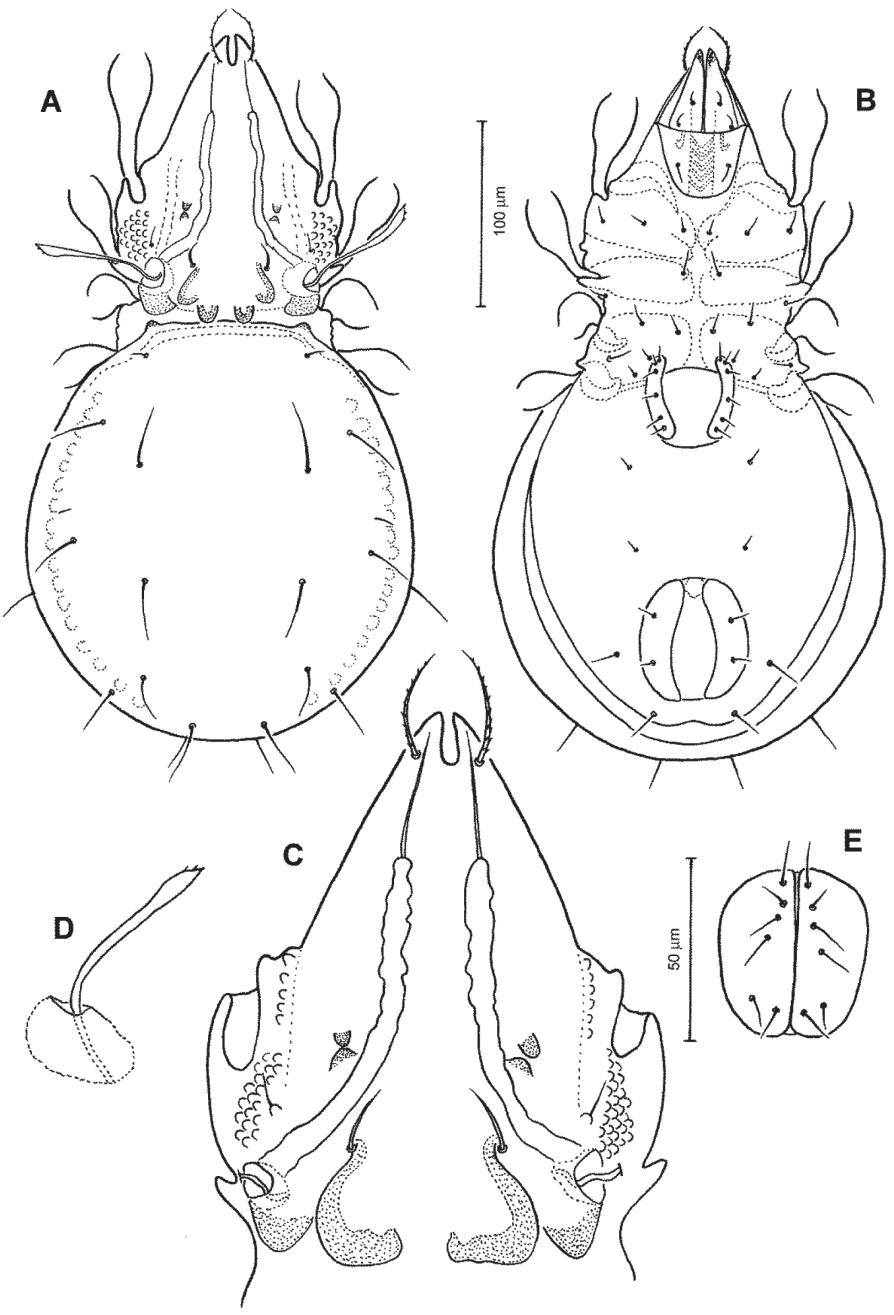
*Conchogneta traegardhi* (Forsslund, 1947). **A** Dorsal view of idiosoma **B** Ventral view of idiosoma **C** Prodorsum **D** Sensillus and bothridium, lateral view **E** Genital plate.

**Figure 5. F5:**
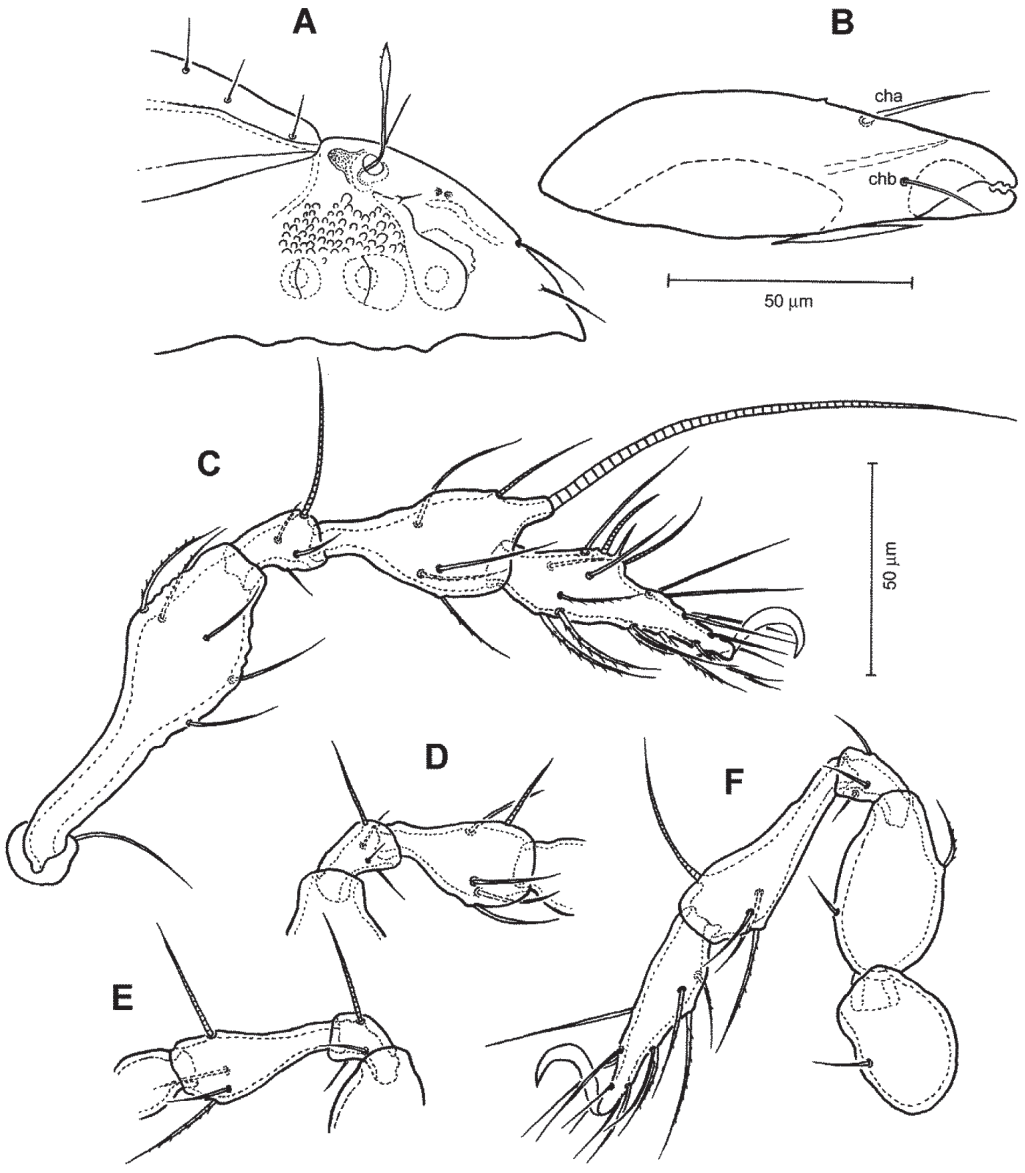
*Conchogneta traegardhi* (Forsslund, 1947). **A** Lateral view of prodorsum and anterior part of notogaster **B** Chelicera, right, antiaxial view **C** Leg I, right, antiaxial view **D** Genu and tibia of leg II, right, antiaxial view **E** Genu and tibia of leg III, right, antiaxial view **F** Leg IV, right, antiaxial view.

**Figure 6.**
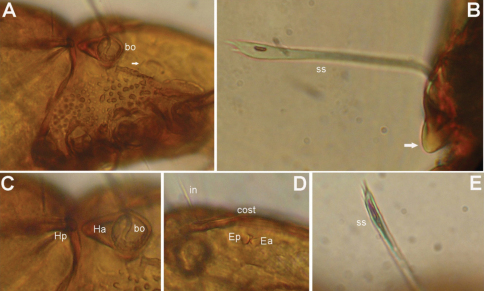
*Conchogneta traegardhi* (Forsslund, 1947). **A** Lateral view of prodorsum, arrow indicates exobothridial seta **B** Sensillus and bothridium, lateral view, arrow indicates postbothridial tubercle *Ha* **C** Humeral region, showing tubercles *Ha* and *Hp* and bothridium **D** Lateral view of part of prodorsum showing prodorsal costula, enantiphysis *E* and interlamellar seta **E** Slight variation of sensillus, lateral view.

*Prodorsum* (Figs 4A, C, D, 5A, 6). Rostrum with deep U-shaped incision reaching level of rostral setal insertion in dorsal view, but distinctly projecting anteroventrally in lateral view (Figs 4A, C, 5A). Rostral seta 28–33 μm long, barbed, curved medially, inserted dorsally on distinct tubercle. Prodorsal costula long, nearly straight, diverging proximally, but parallel anteriorly (Figs 4A, C, 6D). Lamellar seta thin, smooth, 30–34 μm long, straight, inserted at distal end of costula. Interlamellar seta 24–29 μm long, attenuate, smooth; distance between alveoli of *in-in* greater than that of *ro-ro* as viewed in dorsal aspect. Exobothridial seta inserted on distinct tubercle, 10–13 μm in length, smooth, directed anterolaterally (Fig. 4C). Sensillus with relatively long stalk and lanceolate head with two or three barbs; exposed portion of sensillus 70–80 μm in length (Figs 4D, 6B, E). Bothridium large, its opening directed posterolaterally, with large protuberance (tubercle *Ha*) posteriorly (Figs 4C, 5A, 6B, C). Prodorsal enantiophysis *E* well developed, tubercle *Ea* and *Ep* small, same in size, subtriangular in shape (Figs 4A, C, 5A). Interbothridial region with one pair of tubercle, nearly semicircular as viewed in dorsal aspect (Figs 4A, C).

*Notogaster* (Figs 4A, 5A, 6A, C). Oval, slightly narrowed anteriorly, about 1.2 times as long as wide. Anterior margin nearly straight, with large humeral protuberance (tubercle *Hp*; Fig. 6C); posterior margin evenly rounded as viewed in dorsal aspect. Notogastral setae medium long (29–35 μm in length), thin, smooth, not reaching level of insertions of next setal row (Fig. 4A). Lyrifissure *im* well developed; other lyrifissures and opisthonotal gland opening not evident.

*Gnathosoma* (Figs 4B, 5B). Subcapitular mentum slightly wider than long, with minute microtubercles. Hypostomal setae *h*, *a* and *m* medium long, thin, smooth (Figs 4B). Chelicera slender with few slightly sclerotized blunt teeth; seta *cha* about 1.4 times as long as *chb*, both setae smooth; Trägårdh’s organ small, but distinctly developed (Fig. 5B). Palp typical for genus as shown in previous species, palpal setation: 0-2-1-3-10 including solenidion ω of tarsus.

*Epimeral region* (Fig. 4B). Pedotecta I and II well developed, covered with minute granules. Epimeral region nearly smooth; setal formula 3-1-3-3, all setae medium long, smooth. Discidium well developed, projected laterally of leg acetabulum IV.

*Anogenital region* (Fig. 4B). Genital aperture slightly widened anteriorly, anal aperture with same width throughout. Genital, aggenital, anal and adanal setae *ad_3_* medium long; two other adanal setae *ad_1_* and *ad_2_* relatively long, but all ano-genital setae thin, smooth. Adanal lyrifissure not evident.

*Legs* (Fig. 5C–F). Dorsal surface of claws smooth, tibia I with large dorso-distal tubercle overhanging tarsus I. Formula of leg setation (including famulus) I (1-5-3-4-18), II (1-5-3-4-15), III (2-3-2-3-15); IV (1-2-3-3-12); formula of solenidia: I (1-2-2); II (1-1-2); III (1-1-0); IV (0-1-0).

#### Material examined.

Thirty-six specimens: Khuitnii-Am area, Mts Mongol Altai, close to Lake Dayan, District Sagsai, Province Bayan-Ulgii, litter of cool temperate larch forest (*Larix sibiricus* Ledebour, 1833), N48°14', E88°55', elevation 2356 m a.s.l., 18 July 2010; four specimens: same data as above, but from fruticose lichens growing on larch tree barks, 01 August 2010; six specimens: same data as above, but from soil-litter of forest edge, 17 July 2010; three specimens: same data as above, but from soil of steppe, 27 July 2010, Col. B. Bayartogtokh.

#### Remarks.

The characters of specimens studied here from Mongolia are match well with the specimens studied by Forsslund (1947), Golosova (1975), Mahunka (2006) and Weigmann (2006). For the sake of completeness, I provide supplementary descriptions with detailed illustrations.

## Discussion

The members of the oribatid mite genus *Conchogneta* are limited to the Northern Hemisphere and are mainly known from moist, warm soils and litter habitats in temperate regions. However, the diversity of this genus is not high, as most of the species were recorded in Europe, except three species, which have expanded distributions in Asia (three species) and North America (one species). Most species of *Conchogneta* are inhabitants of various type forest litters (beech, birch, fir, spruce, pine, larch), bryophytes (*Hypnum* sp., *Sphagnum* sp.) growing on the forest floor, in cave or as epyphytes on hazel trees, as well as soils in river valleys, moor, oligotrophic bogs, floodland assemblages (Weigmann and Kratz 1982, Beck and Woas 1991; Huhta and Niemi 2003, Sidorchuk 2009; Toluk and Ayyildiz 2009).

The genus is represented in Mongolia with only two species studied here, and one of them, *Conchogneta traegardhi* is the most widely distributed species of *Conchoneta*, which is known from Palaearctic and Nearctic regions. It should be noted here that Subías (2010) removed this species from *Conchogneta* and included it in the genus *Autogneta* Hull, 1916, without any commentary. However, the structure of the sensilli speaks against the inclusion of *Conchogneta traegardhi* in *Autogneta*, as the other species of *Autogneta* have clavate or capitate sensilli in contrast to lanceolate or fusiform sensilli in *Conchogneta traegardhi*, which is typical of *Conchogneta* (Weigmann 2006). Moreover, according to Grandjean (1963), the genera *Autogneta* and *Conchogneta* generally differ in their ontogeny, with the juvenile stages of latter exhibiting spatulate-pateriform setae on both the prodorsum and notogaster.

*Conchogneta traegardhi* is known to be a sylvicolous species, widely distributed in Eurasia and North America, but it is nowhere common (Mahunka 2006). However, it is one of the dominating species in the Mongol Altai Mountains, where I found it abundantly in the litter of the interior of larch forests. It occurs with up to 28 individuals per 125 cm^3^ of soil-litter samples collected in the forest interior, but was rarely found in the forest edge or steppe soils, where less than 4 individuals per sample were found. The livestock primarily grazes in the steppe, but also forest margins and less intensively the interior of forests are utilized for pasture. Additionally, were also collected a few individuals of *Conchogneta traegardhi* from the fruticose lichens (*Xanthoria candelaria*, *Rhizoplaca chrysolenca*, *Parmeliopsis ambigua*) growing at the trunk base of larch trees. Most specimens of the present species had food in their gut and food boluses primarily contained fungal hyphae. Heggen (2010) revealed *Conchogneta traegardhi* as an inhabitant of the lower zones of alpine regions in Fennoscandia, but did not find it in the higher alpine zones. Therefore, Heggen (2010) concluded that the distribution of *Conchogneta traegardhi* might be limited by altitude. However, this species is abundantly occurred in the high alpine zone of the Mongoli Altai Mountains at elevation of more than 2300 m above sea level. Therefore, I suggest that the upper limit distribution of this species depends more on the occurrence of forests than on elevation (and, with it, temperature) itself.

The second species found in Mongolia, *Conchogneta glabrisensillata* sp. n. is quite rare, and was only found in a few samples of single valley out of six studied valleys in the eastern tributaries of Lake Hövsgöl, where many soil, litter and lichen samples were investigated. The valley is a broad, flat valley with steppe vegetation covering the valley bottom and south-facing slopes of mountains on the north side of the valley. The extensive larch forests cover the north facing slopes of the mountains. The valley floor consists of sandy soils and the river sediment is also very sandy. However, the forest floor has a fairly thick litter horizon with black humus rich soil mixed with mosses and lichens. There is relatively heavy grazing with indications of excessive grazing on the south facing slopes, but less grazing pressure in the forest. The new species was collected from litter of a larch forest, and the area is very cold, but one of the moistest regions within Mongolia. While currently known only from forest litter at the type locality, *Conchogneta glabrisensillata* sp. n. probably has a restricted geographic distribution and ecological niche in cold areas.

In the regular update of the checklist of world oribatid mites, Subías (2006) treated the type species of *Conchogneta*, *Conchogneta dalecarlica* (Forsslund, 1947), as a junior synonym of *Conchogneta willmanni* (Dyrdowska, 1929). Indeed these two species are similar to each other, especially in the widely spaced prodorsal costulae. However, not only these two species, but also several other members of *Conchogneta*, including *Conchogneta inundata*, *Conchogneta weigmanni* and *Conchogneta willmanni herzegowinensis*, share the costulae laterally placed on prodorsum. The other species have closely placed costulae, which are situated along the center of prodorsum. Thus, all species of *Conchogneta* can be classified into two groups in respect to their structure of prodorsal costulae.

When he synonymized *Conchogneta dalecarlica* with *Conchogneta willmanni*, Subías (2006) did not provide any commentary or justification. In contast to Subías (2006), *Conchogneta dalecarlica* and *Conchogneta willmanni* are treated here as different species, because they differ in the structure of the sensilli and the prodorsal costulae (Mahunka 2006, Akrami 2008). This view agrees with that of Woas (1986), Weigmann (2006), Toluk and Ayyildiz (2009).

The following key can be used to identify adults of all known species of *Conchogneta*.

### World-wide key to the adults of Conchogneta

**Table:** 

1	Prodorsal costula widely spaced from each other, placed laterally on prodorsum, strongly converging anteriorly	2
–	Prodorsal costula closely placed to each other, situated along center of prodorsum, nearly parallel or slightly converging anteriorly	7
2	Sensillis setiform or very slightly dilated distally	3
–	Sensillus lanceolate or pectinate	5
3	Notogastral setae medium long, not reaching alveoli of next setal row; interlamellar seta short	4
–	Notogastral setae long, reaching alveoli of next setal row; interlamellar seta long	*Conchogneta wilmanni herzegowinensis* Willmann, 1941
4	Sensillus with long ciliae; costula very widely spaced from each other	*Conchogneta iranica* Akrami, 2008
–	Sensillus smooth; costula relatively close to each other	*Conchogneta inundata* (Winkler, 1957)
5	Prodorsum with one pair of basal tubercles; costula thin, without lateral oval field	6
–	Prodorsum with two pairs of basal tubercles; costula very thick, with lateral oval field	*Conchogneta weigmanni* Mahunka, 2007
6	Sensillus very long, its head bifurcate	*Conchogneta willmanni* (Dyrdowska, 1929)
–	Sensillus relatively short, its head pectinate or well pilose	*Conchogneta dalecarlica* (Forsslund, 1947)
7	Distal part of costula not dilated; sensillus lanceolate, distally covered with few short barbs or smooth; interlamellar seta smooth; body length smaller than 430 µm	8
–	Distal part of costula dilated; sensillus baciliform, distally covered with many short barbs; interlamellar seta barbed; body length greater than 500 µm	*Conchogneta vasiliorum* Mahunka, 2006
8	Prodorsal tubercles *Ea* much smaller than *Ep*; sensillus smooth; anterior part of costula not straight, but slightly rounding	*Conchogneta glabrisensillata* sp. n.
–	Prodorsal tubercles *Ea* and *Ep* small, same in size; sensillus with few, but distinct barbs; anterior part of costula nearly straight	*Conchogneta traegardhi* (Forsslund, 1947)

## Supplementary Material

XML Treatment for Conchogneta glabrisensillata

XML Treatment for Conchogneta traegardhi
